# Supplementary material for: The basolateral amygdala-anterior cingulate pathway contributes to depression-like behaviors and comorbidity with chronic pain behaviors in male mice
Source: Nat Commun. 2023 Apr 17;14:2198. doi: 10.1038/s41467-023-37878-y (PMC10110607; doi:10.1038/s41467-023-37878-y)
Supplement: Supplementary file 1 — Supplementary Information [file 41467_2023_37878_MOESM1_ESM.pdf]

The basolateral amygdala-anterior cingulate pathway contributes to depression-like behaviors and comorbidity with chronic pain behaviors in male mice

Supplementary information

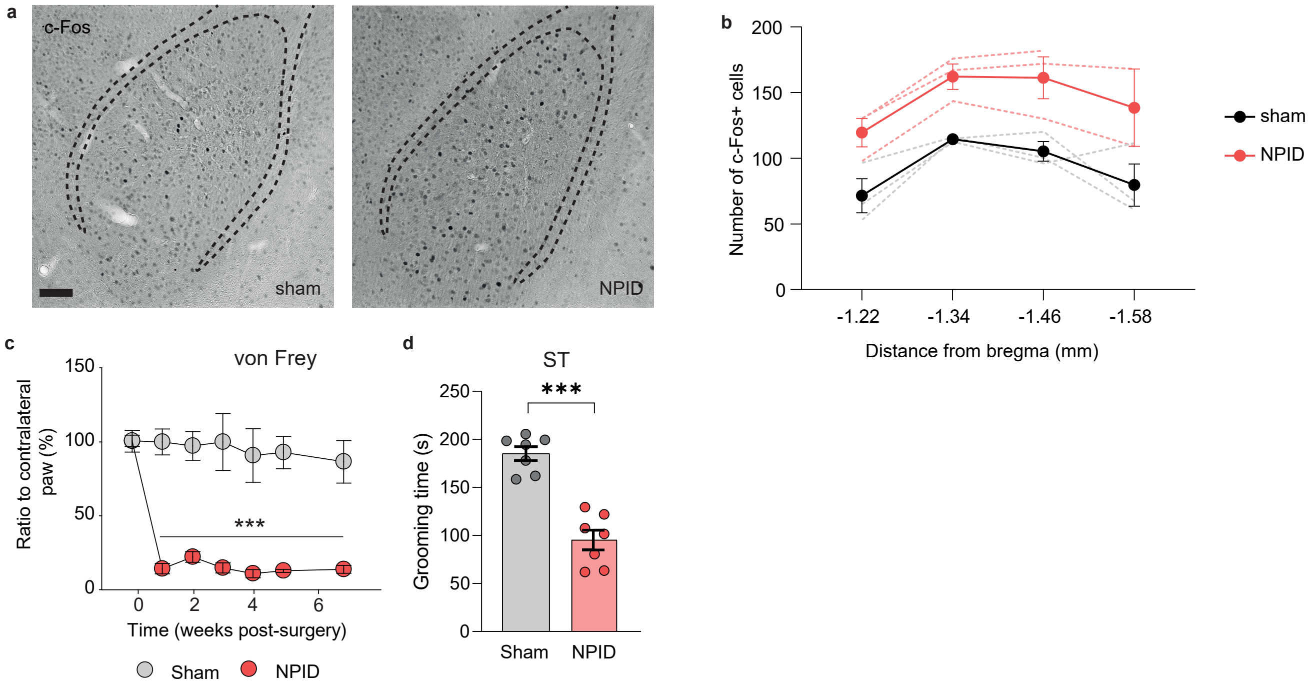

Supplementary Figure 1.

a. Representative images showing c-Fos immunoreactivity in the left (contralateral to the nerve injury) BLA of sham (left panel) and NPID (right panel) animals at 8 weeks PO. Scale bar=100µm. **b.** The number of c-Fos positive cells was increased in the BLA in NPID animals (sham: n=3 mice; NPID: n=3 mice; F(1,4)=15.44; p=0.017, dotted traces=individual responses). **c-d.** Peripheral nerve injury induced an ipsilateral long-lasting mechanical hypersensitivity (c; sham: n=7 mice; cuff: n=7 mice; F(6,72)=6.2629; p<0.0001; post-hoc weeks 1-7 p<0.05) and decreased grooming behavior in the splash test (d; sham: n=7 mice; 185.21±19.07; NPID: n=7 mice; 95.21±27.11; p=0.00001). Data are mean ± SEM. 2-Way ANOVA (anterioposteriority x Surgery: c-Fos quantification); 2-Way ANOVA repeated measures (Time x Surgery; VF); one-tailed Mann-Whitney test (FG, c-Fos quantification, Splash test). PO, post-operative; ST, Splash test; VF, von Frey filament test. Source data are provided as a Source Data file

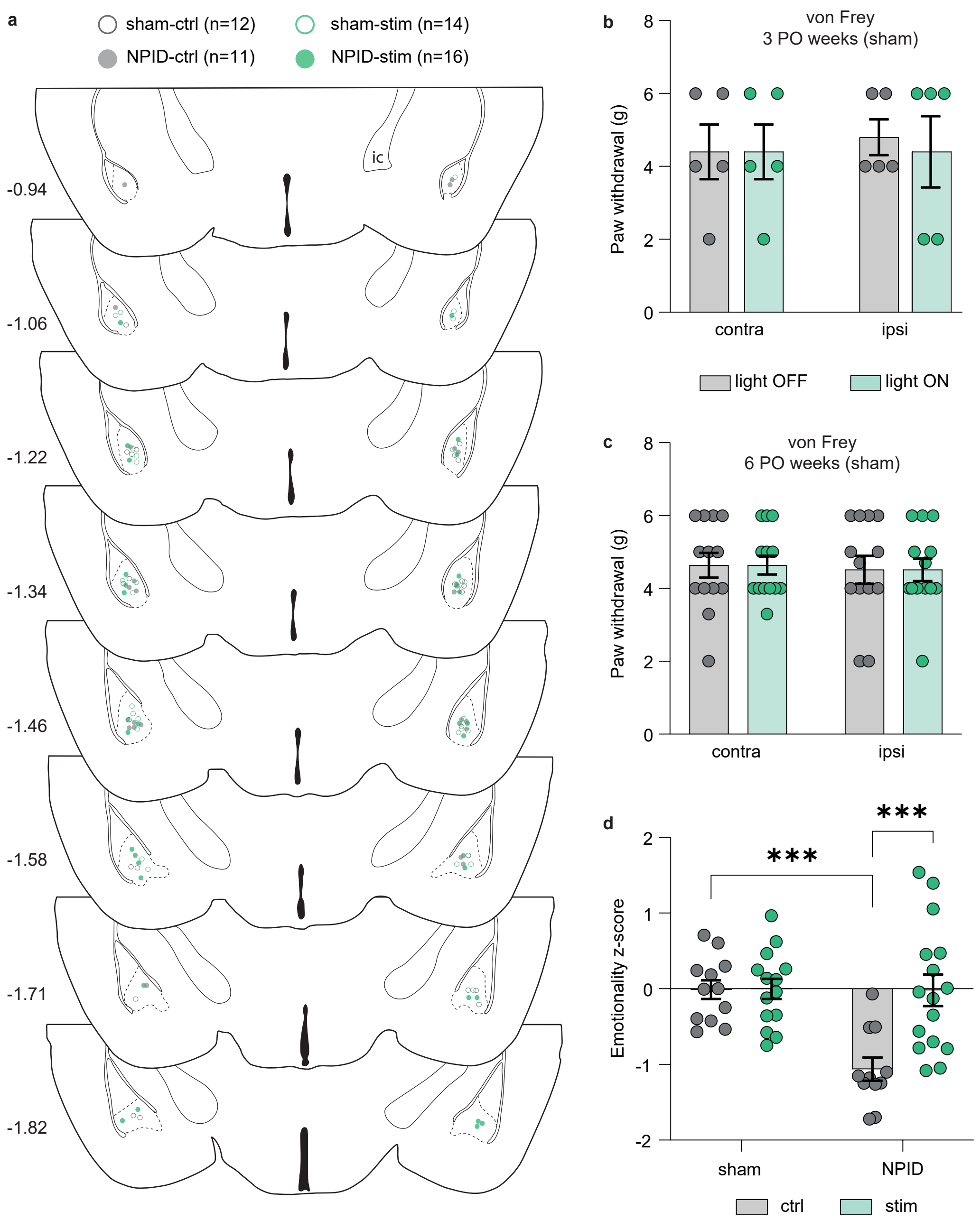

Supplementary Figure 2.

a. Schematic diagrams illustrating the localization of the injection sites of AAV5-CaMKIIa-eArchT3.0-EYFP in the BLA based on the Allen Mouse Brain Atlas. At 3 (**b**) or 6 (**c**) weeks after peripheral nerve injury, mechanical threshold was not altered by optogenetic inhibition of the BLA-ACC pathway in sham animals (ipsilateral vs contralateral paw; 3 PO weeks (n= 5 mice), $F(1,4)=0.12$; $p=0.75$; 6 PO weeks (n=13 mice), $F(1,12)=0.19$; $p=0.67$; light-off vs light-on; 3 PO weeks $F(1,4)=0.12$; $p=0.75$; 6 PO weeks $F(1,12)=0.00$; $p>0.99$). **d.** NPID animals had decreased emotionality z-score which were reversed by optogenetic inhibition of the BLA-ACC pathway ($F(1,49)=9.274$; $p=0.003$; post-hoc: sham-ctrl (n=12 mice)>NPID-ctrl (n=11 mice); $p<0.001$; NPID-ctrl (n=11 mice)<NPID-stim (n=16 mice); $p<0.001$; sham-ctrl (n=12 mice)=sham-stim (n=14 mice); $p>0.05$). Data are mean±SEM. ***$p<0.001$. Two-way ANOVA repeated measures (von Frey), two-way ANOVA (surgery x stimulation; Emotionality z-score). Source data are provided as a Source Data file

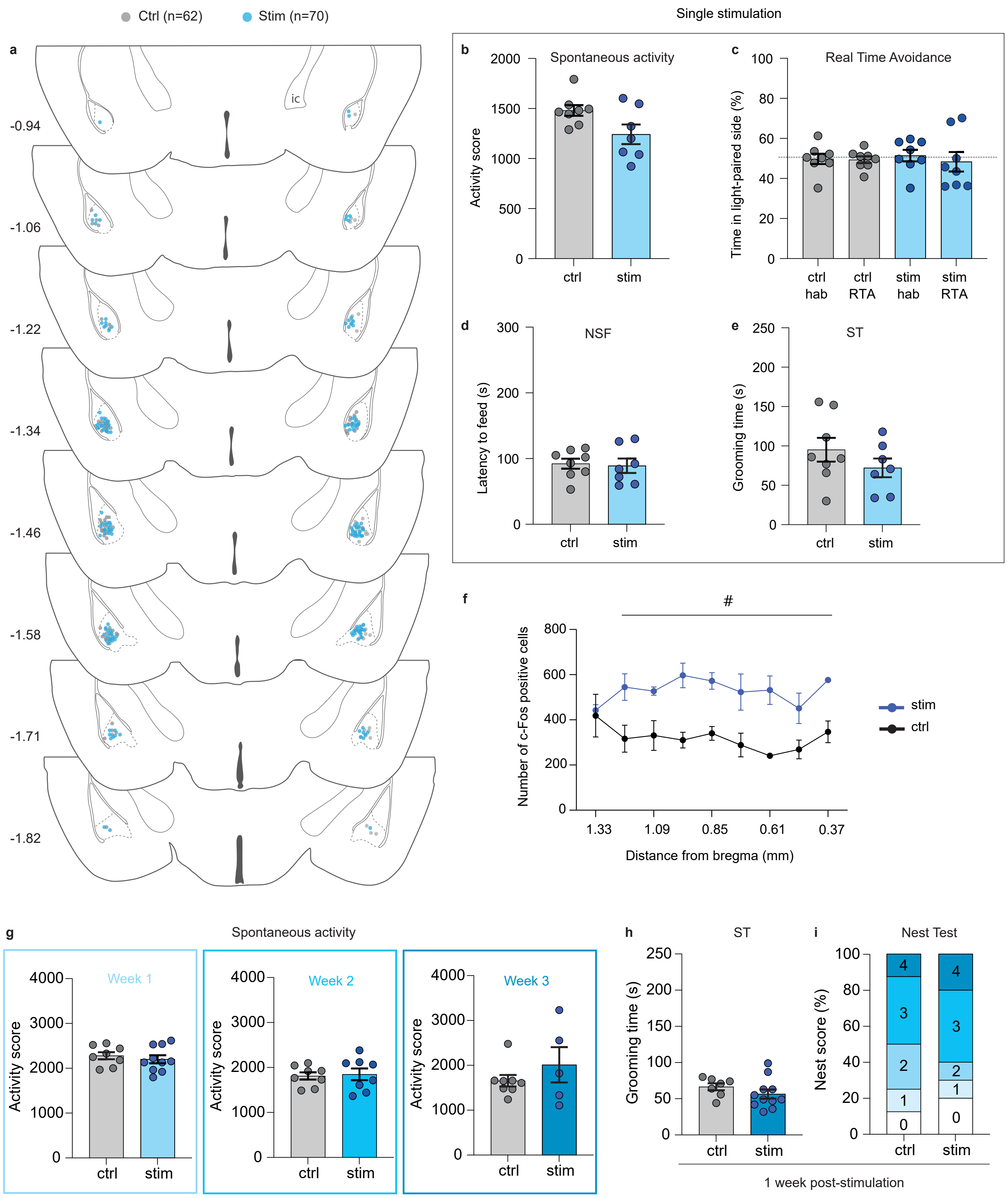

Supplementary Figure 3.

a. Schematic diagrams illustrating the localization of the injection sites of AAV5-CaMKIIa-ChR2(H134R)-EYFP in the BLA based on the Allen Mouse Brain Atlas. **b-c.** Single activation of the BLA-ACC pathway did not alter spontaneous locomotor activity (b; ctrl n=8 mice; 1480±53.37; stim n=7 mice; 1329±85.36; p=0.15), or induce avoidance in the real time avoidance test (c; RTA, ctrl: n=8 mice; stim: n=8 mice; F(1, 14)=0.006; p=0.94). **d-e.** Single activation of the BLA-ACC pathway did not change anxiety-like behaviors in the NSF (d; ctrl n=8 mice; 92.25±7.57; stim n=7 mice; 89.14±10.97; p=0.82), or the grooming time in the ST (e; ctrl n=8 mice; 95.25±15.13; stim n=7 mice; 72.14±11.85; p=0.26). **f.** Optogenetic activation of the BLA-ACC pathway increased c-Fos immunoreactivity in whole ACC (24a/24b) (ctrl: n=4 mice; stim: n=4 mice; F(1, 6) =17.24; p=0.006). **g.** Repeated activation of the BLA-ACC pathway did not change spontaneous locomotor activity (3 stim: ctrl: n=8 mice; 2281±78.89; stim: n=10 mice; 2202±88.73; p=0.53; 6 stim: ctrl: n=8 mice; 1812±80.24; stim: n=8 mice; 1849±129.3; p=0.81; 9 stim: ctrl: n=8 mice; 1656±128.0; stim: n=5 mice; 2011±393.4; p=0.33). **h-i.** One week after the ninth stimulation, no further deficits in grooming (h; ctrl: n=7 mice; 66.57±4.86; stim: n=11 mice; 56.55±6.22; p=0.27) or nesting behaviors (i; ctrl: n=7 mice; stim: n=11 mice; Chi-square=1.469; p=0.83) was observed in stimulated animals. Data are mean ± SEM. #=main effect. Two-sided unpaired t-test (Spontaneous activity, NSF, ST); chi-square test for trend (Nest test); Two-Way ANOVA repeated measures (RTA: Time point x Stimulation; c-Fos immunohistochemistry: Stimulation x Anteroposteriority) Source data are provided as a Source Data file

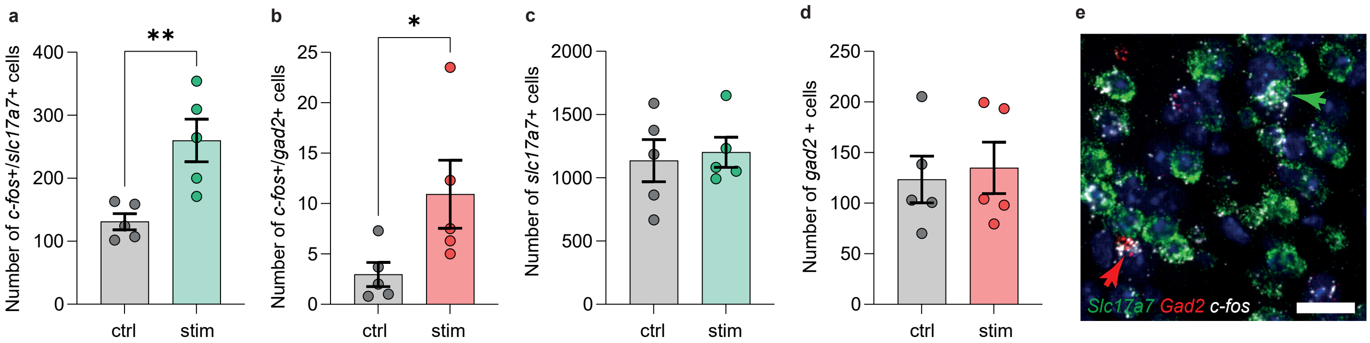

Supplementary Figure 4.
a-d. mRNA in situ hybridization (RNAscope) revealed that optogenetic activation of the BLA-ACC pathway increased the number of c-fos+/Slc17a7+ cells (panel a; ctrl; n=5 mice; 131.0±12.81; stim: n=5 mice; 259.9±33.83 p=0.007) and the number of c-fos+/Gad2+ cells in the ACC (panel b; ctrl: n=5 mice; 2.96±1.20 stim: n=5 mice; 10.92±3.38 p=0.032). The number of Slc17a7+ (panel c; ctrl: n=5 mice; 1136±167.0 stim: n=5 mice; 1202±119.1, p=0.84) and Gad2+ cells (panel d; ctrl: n=5 mice; 123.5±23.15 stim: n=5 mice; 134.9±25.49 p>0.99) remained unaffected by optogenetic stimulation of the BLA-ACC pathway. **e.** Close-up image showing co-localization between c-fos and Slca17a7 (green arrow) and between c-fos and Gad2 (red arrow). Scale bar=20µm. Data are mean±SEM. *p<0.05; **p<0.01 One-sided mann-Whitney test (Slc17a7, Gad2, c-fos/Slc17a7 and c-fos/Gad2 mRNA quantification). Source data are provided as a Source Data file

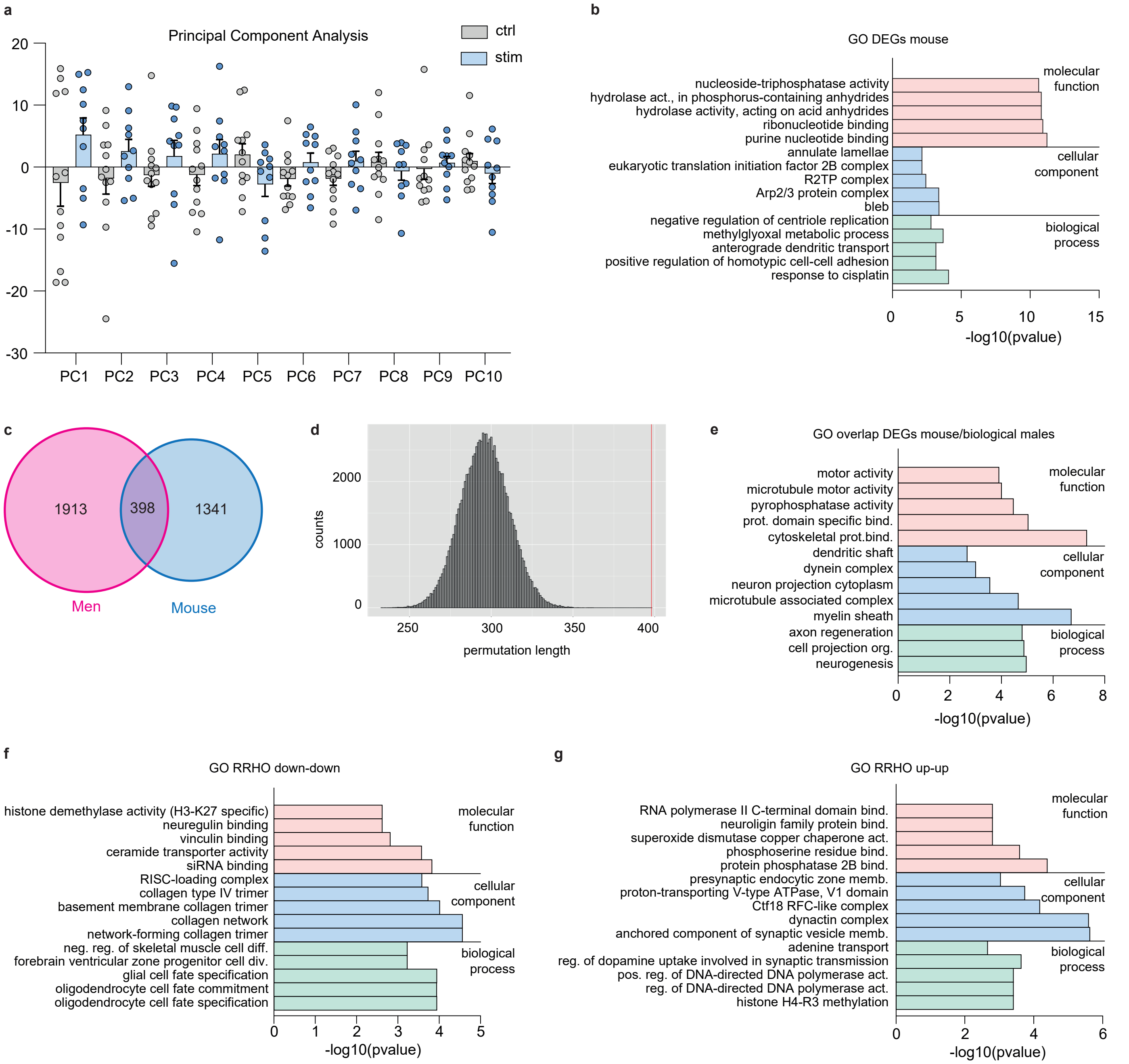

Supplementary Figure 5.

a. Principal Components Analysis showed robust differences between control (n=12 mice) and stimulated (n=10 mice) animals at the whole genome level. **b.** GO enrichment (biological process, cellular component and molecular function) of the 2611 differentially expressed genes (DEG, nominally significant) in stimulated animals. **c.** Among the 1739 mouse DEG with a human orthologue, 398 (29.6%) were also differentially expressed in men. **d.** Permutation analysis of the mouse/human intersection depicted in panel c (n=100,000 permutations). Histograms indicate numbers of expected intersections (y-axis) of a given size (x-axis), while the red line depicts the observed intersection (hypergeometric test, p=6.6E-12). **e.** GO enrichment (biological process, cellular component and molecular function) of the 398 differentially expressed genes in both mice and men. **f-g.** GO enrichment (biological process, cellular component and molecular function) of the genes commonly down-regulated (f) and up-regulated (g) in mice and men. Data are mean±SEM. Hypergeometric test with Benjamini and Hochberg correction for multiple testing (GO). Source data are provided as a Source Data file

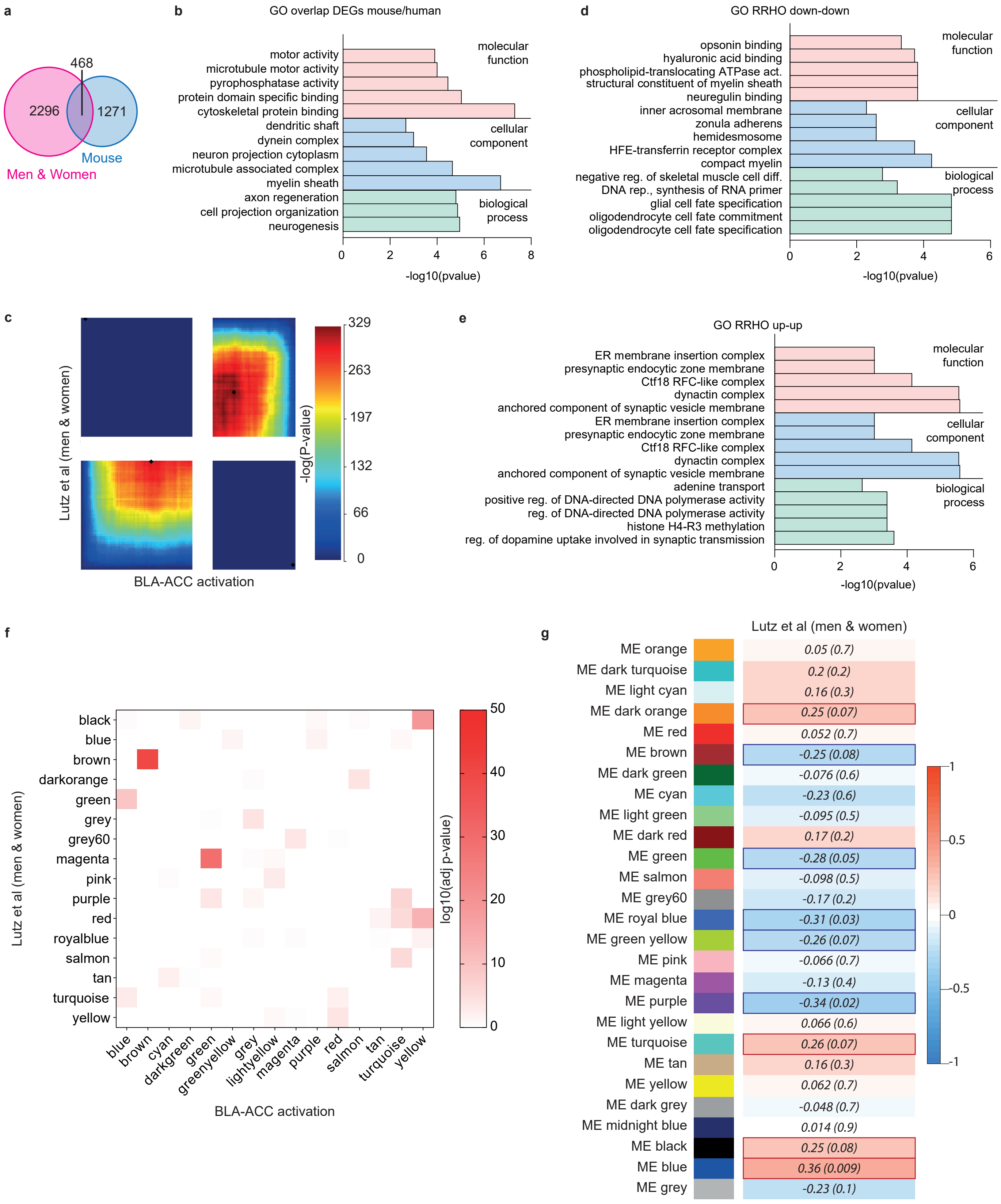

Supplementary Figure 6.

a. Among the 1379 orthologous genes differentially expressed in mice, 468 (34.9%) were also differentially expressed in humans (men and women). **b.** GO enrichment (biological process, cellular component and molecular function) of the 468 differentially expressed genes in both mice and humans. **c.** Rank-Rank Hypergeometric Overlap (RRHO2) identified shared transcriptomic changes in the ACC across mice and humans (men and women) as a function of optogenetic stimulation (mouse) or a diagnosis of major depressive disorder (MDD). Levels of significance for the rank overlap between humans and mice are color-coded, with a maximal one-sided Fisher's Exact Test p<1.0E-328 for up-regulated genes (bottom-left panel) and maximal FET p<1.0E-311 for down-regulated genes (upper-right panel). **d-e.** GO enrichment (biological process, cellular component and molecular function) of the genes commonly down-regulated (d) or up-regulated (e) in mice and humans. **f.** Heatmap representing the level of significance of overlaps between mouse and human gene modules (measured using the FET). The highest overlap (p=3.52E-52) was obtained for the human/brown and mouse/brown modules. **g.** WGCNA was used to analyze network and modular gene co-expression in the human ACC. The tables depict associations between individual gene modules and MDD diagnosis in humans. Each row corresponds to correlations and p-values obtained against each module's eigengene. Hypergeometric test with Benjamini and Hochberg correction for multiple testing (GO).

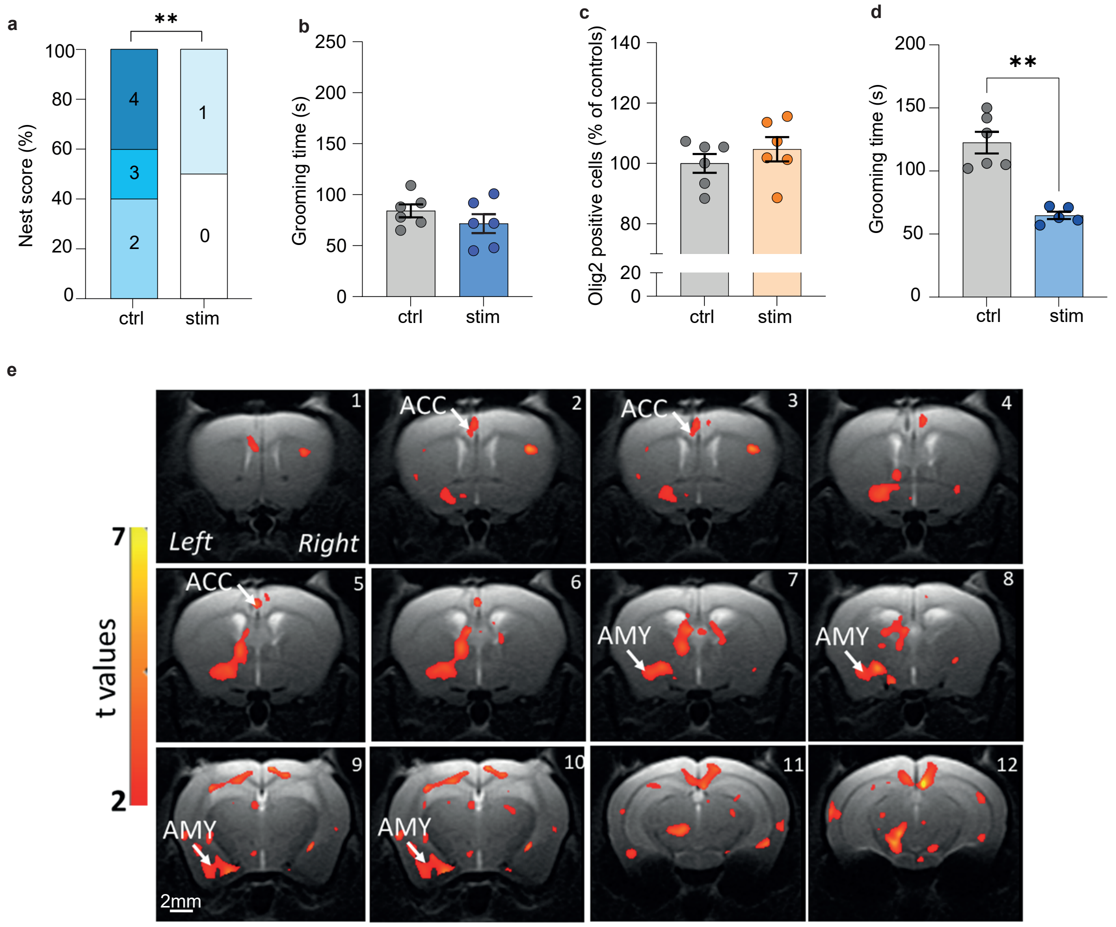

Supplementary Figure 7.

a. Repeated activation of the BLA-ACC pathway decreased nest quality in stimulated animals (ctrl n=5 mice; stim n=6 mice; Chi-square=8.317; p=0.0039). **b.** One week after the ninth stimulation, no further deficit in grooming was observed in stimulated animals (ctrl: n=6 mice; 84.17±6.38; stim: n=6 mice; 71.67±9.21; p=0.29). **c.** Quantification of Olig2+ cells showed that one week after that last stimulation of the BLA-ACC pathway, the number of Olig2+ cells in the ACC returned to control level (d: ctrl: n=6 mice; 100.00±3.115; stim: n=6 mice; 104.7±4.012; p=0.15). **d.** Repeated activation of the BLA-ACC decreased grooming time in stimulated animals (ctrl n=6 mice; 122.5±8.55; stim n=5 mice; 64.80±2.90; p<0.002). **e.** Representative coronal MRI images showing brain areas (in red) with significant positive correlation between fractional anisotropy values and grooming time (ctrl n=8 mice; stim=7 mice; p<0.001; uncorrected). Microstructural changes reflected by low fractional anisotropy (FA) along the left BLA-ACC pathway (see Fig. 6d) correlated with low grooming time in stimulated animals. Data are represented as mean±SEM. **p<0.01. chi-square test for trend (Nest test); One-sided (Olig2 quantification) and two-sided (ST) Mann-Whitney test. Source data are provided as a Source Data file.

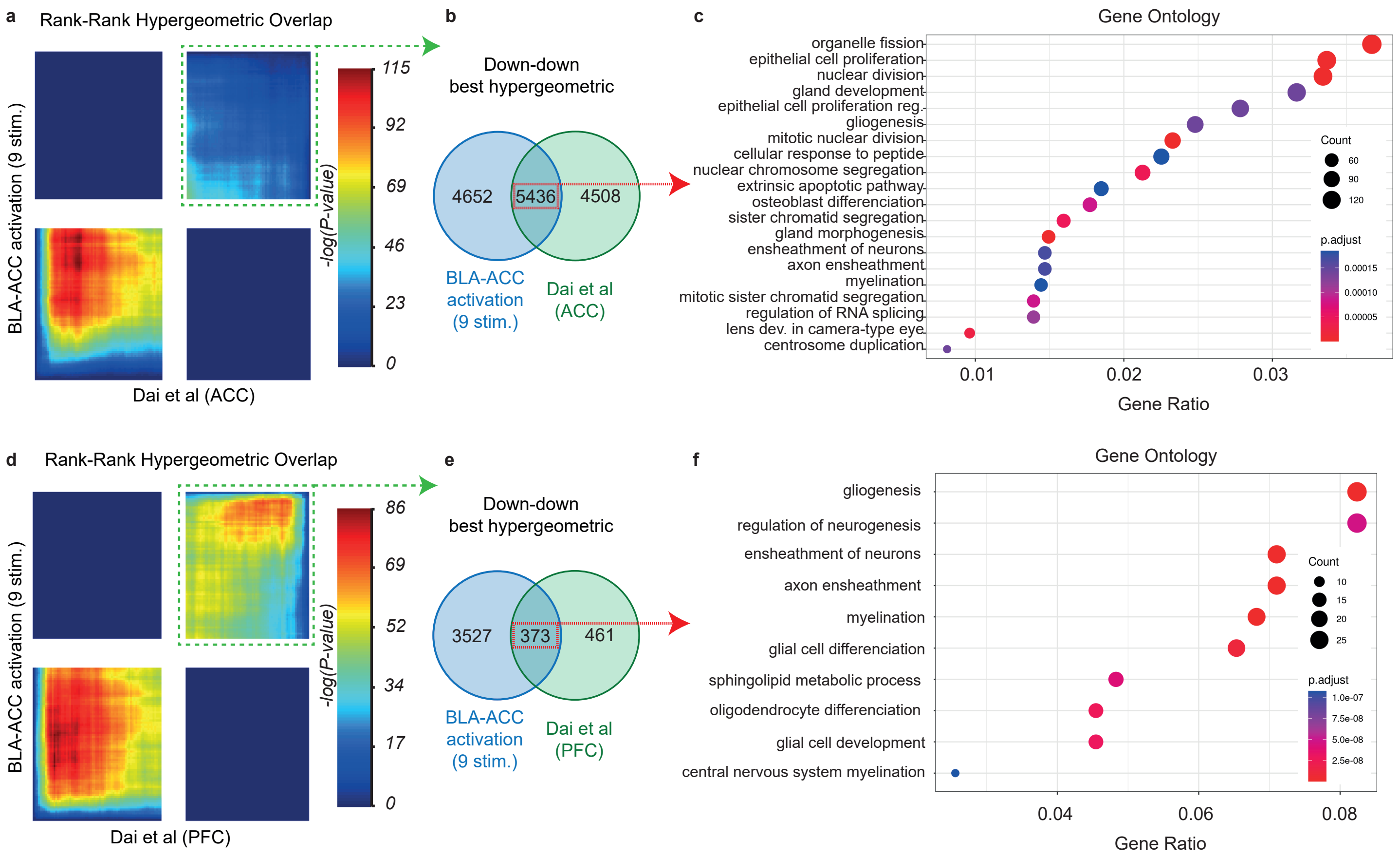

Supplementary Figure 8

a. Rank-Rank Hypergeometric Overlap (RRHO2) identified shared transcriptomic changes in the ACC across our optogenetic BLA-ACC paradigm and the spared nerve injury (SNI) model of neuropathic pain (Dai et al. 2022). Levels of significance for the rank overlap are color-coded, with a maximal one-sided Fisher's Exact Test p<1.0E-115 for up-regulated genes (bottom-left panel) and maximal FET p<1.0E-55 for down-regulated genes (upper-right panel). **b.** Venn Diagram of the best overlap among down-regulated genes in the ACC of BLA-ACC stimulated mice and SNI model. **c.** GO enrichment of genes commonly down-regulated in the ACC of BLA-ACC stimulated mice and SNI model, generated using clusterProfiler. **d.** Rank-Rank Hypergeometric Overlap (RRHO2) identified shared transcriptomic changes in the ACC in our optogenetic paradigm and the PFC in SNI. Levels of significance for the rank overlap are color-coded, with a maximal one-sided Fisher's Exact Test p<1.0E-86 for up-regulated gene (bottom-left panel) and maximal FET p<1.0E-70 for down-regulated genes (upper-right panel). **e.** Venn Diagram of the best overlap among up-regulated genes in the ACC of BLA-ACC stimulated mice and in the PFC of SNI mice. **f.** GO enrichment of genes commonly down-regulated in the ACC of BLA-ACC stimulated mice and SNI model, generated using clusterProfiler. GO: one-sided Fisher's exact test with Benjamini and Hochberg correction.

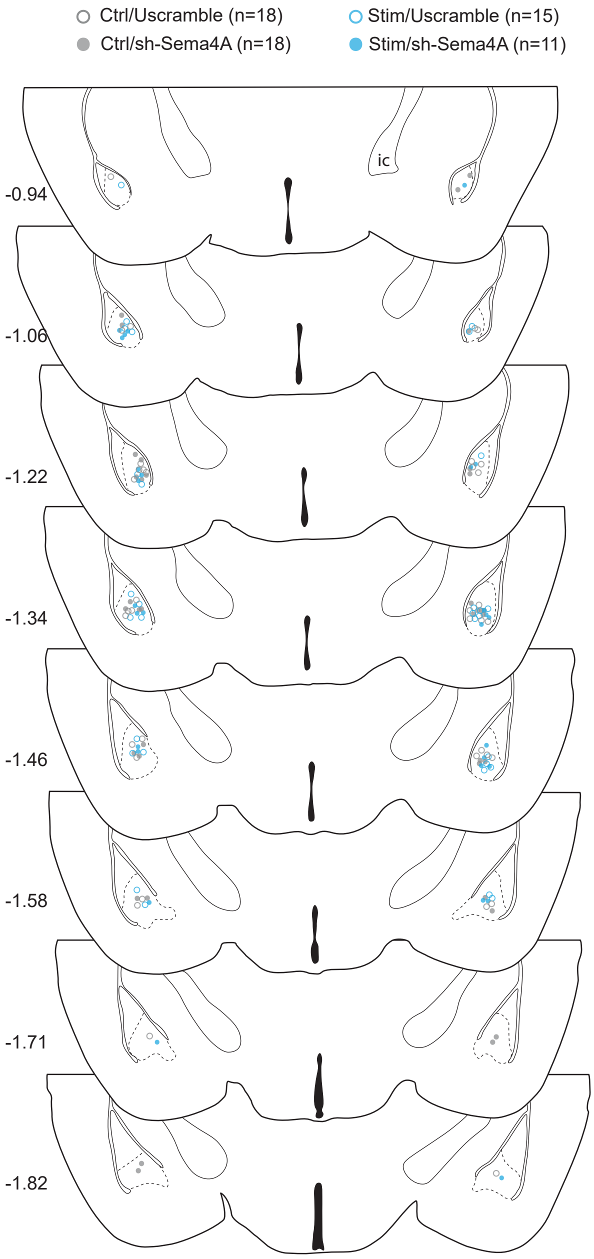

Supplementary Figure 9.

Schematic diagrams illustrating the localization in the BLA of injection sites of the AAV5-CaMKIIa-ChR2(H134R)-EYFP vector, based on the Allen Mouse Brain Atlas.

Supplementary Table 1

Description of human and mouse cohorts used for transcriptomic analysis.

Human	MDD (n=26)	Controls (n=24)
Age (Years)	42,2 ± 2.85	46.2 ± 4.4
Sex (M/F)	19/7	19/5
RIN	6.83 ± 0.15	6.86 ± 0.12
Mouse	Stimulated (n=10)	Controls (n=12)
Batch (1/2)	4/6	6/6
Sex (M/F)	10/0	12/0
RIN	9.40 ± 0.28	9.44 ± 0.15

Supplementary Table 2

Gene ontology analysis (Two-sided Fisher Exact Test) of the five mouse modules significantly associated with optogenetic stimulation and conserved in humans.

module	enrichmentP	BonferoniP	termOntology	termName
blue	1,49E-08	0,000269653	CC	preribosome
blue	1,55E-06	0,028138839	BP	RNA processing
blue	3,32E-06	0,060160709	CC	cytoplasmic vesicle
blue	3,82E-06	0,069182237	CC	intracellular vesicle
blue	8,64E-06	0,156507228	CC	clathrin-coated vesicle membrane
blue	9,72E-06	0,175981124	CC	clathrin-coated vesicle
blue	9,94E-06	0,179982376	CC	protein-containing complex
blue	1,08E-05	0,195746883	BP	mRNA processing
blue	1,19E-05	0,215232033	MF	RNA binding
blue	1,25E-05	0,226475177	MF	ATP-dependent protein binding
brown	2,89E-10	5,23E-06	BP	myelination
brown	4,61E-10	8,36E-06	BP	axon ensheathment
brown	1,39E-08	0,000252159	CC	myelin sheath
brown	7,71E-08	0,001395487	BP	oligodendrocyte differentiation
brown	8,93E-08	0,001616898	BP	glial cell differentiation
brown	9,37E-08	0,001697005	BP	gliogenesis
brown	1,80E-07	0,003260951	BP	nervous system development
brown	1,87E-07	0,003391081	CC	cell periphery
brown	3,49E-07	0,006325485	BP	neurogenesis
brown	1,21E-06	0,02197177	CC	plasma membrane
greenyellow	0,00015471	1	BP	neuronal action potential
greenyellow	0,00036253	1	MF	ion channel activity
greenyellow	0,00059938	1	MF	channel activity
greenyellow	0,000614	1	MF	passive transmembrane transporter activity
greenyellow	0,00069401	1	BP	action potential
greenyellow	0,00084187	1	MF	ion transmembrane transporter activity
greenyellow	0,00094943	1	BP	trigeminal nerve structural organization
greenyellow	0,00103757	1	MF	cation channel activity
greenyellow	0,00126358	1	CC	calyx of Held
greenyellow	0,00141493	1	BP	trigeminal nerve development

magenta	0,0002393	1	MF	ubiquitin-like protein-specific protease activity
magenta	0,00082264	1	CC	nucleoplasm
magenta	0,00118341	1	MF	nucleic acid binding
magenta	0,00218795	1	BP	protein desumoylation
magenta	0,00228823	1	MF	cysteine-type peptidase activity
magenta	0,00255046	1	CC	cytoplasmic stress granule
magenta	0,00349927	1	CC	nucleus
magenta	0,0038129	1	CC	nuclear lumen
magenta	0,0045933	1	BP	double-strand break repair via break-induced replication
magenta	0,00480606	1	BP	lung alveolus development
yellow	1,90E-15	3,44E-11	BP	mitochondrial translational termination
yellow	5,13E-15	9,28E-11	BP	translational elongation
yellow	5,24E-15	9,49E-11	CC	mitochondrial inner membrane
yellow	1,05E-14	1,90E-10	BP	mitochondrial translational elongation
yellow	1,51E-13	2,74E-09	BP	translational termination
yellow	2,07E-13	3,74E-09	BP	mitochondrial translation
yellow	2,58E-13	4,67E-09	CC	mitochondrial envelope
yellow	3,71E-13	6,72E-09	CC	organelle inner membrane
yellow	9,12E-13	1,65E-08	CC	mitochondrial membrane
yellow	1,54E-12	2,79E-08	BP	regulation of cellular amino acid metabolic process

Supplementary Table 3

Primers sequences used for RT-qPCR (Fluidigm) in Fig.6h-i.

Gene name	Sequence
B2m	F TGCTACGTAACACAGTTCCACC<br>R: TCTGCAGGCGTATGTATCAGTC
Gapdh	F: TGGCCTCCAAGGAGTAAGAAAC<br>R: TGGGATGGAAATTGTGAGGGAG
Actb	F: ATCAGCAAGCAGGAGTACGATG<br>R: GGTGTAAAACGCAGCTCAGTAAC
Gusb	F: TACCGACATGAGAGTGGTGTTG<br>R: TAATGTCAGCCTCAAAGGGGAG
Plp1	F: AGCAAAGTCAGCCGCAAAAC<br>R: TGAGAGCTTCATGTCCACATCC
Ermn	F: AGAGAACCTCTTCGTTGTTCACC<br>R: TTGCTGGGCAGTTCTTTCCTTC
Aspa	F: CACTTCTAACATGGGTTGCACTC<br>R: AAACAGAGCAGGGTAATGGAGC
Ugt8	F: TGAAGGAGAGCTGTATGATGCC<br>R: TCCGTCATGGCGAAGAATGTAG
Mal	F: ATTACCATGAAAACATCGCCGC<br>R: TTAATGGGGAAGATGGGCTGAC
Mog	F: CATAAAGATGGCCTGTTTGTGGAG<br>R: CCCTGGTCCTATCACTCTGAATTG
Mbp	F: ATCGGCTCACAAGGGATTCAAG<br>R: TATATTAAGAAGCCGAGGGCAGG
Lingo1	F: CAACAAGACCTTCGCCTTCATC<br>R: TGCTTTGTGTTGCCTTTGCC
Sema4a	F: AGCCATGTGGTCATGTATCTGG<br>R: TGAATCTCCTCCACGAGATAAGC

Supplementary Note 1: Analysis codes

RRHO2

```
>Library(RRHO2)

> DATA1=input_RRHO2_S19219 #mouse ACC differential expression data organized as follow: column1: geneID, colone2: log2fc, column 3: pvalue

>DATA2=hACC_men_only # men ACC differential expression data organized as follow: column1: geneID, colone2: log2fc, column 3: pvalue

#create df containing only genes expressed in both species.
>merge_DATA1_DATA2 <- merge(DATA1, DATA2, by.x = 1, by.y= 1)
>DATA1.2 <- merge_DATA1_DATA2[,c(1:3)]
>DATA2.2 <- merge_DATA1_DATA2[,c(1,4,5)]

#Rank genes according to their pvalue and log2fc
>DATA1_ordered <- cbind(data.frame(DATA1.2$geneID,stringsAsFactors =TRUE), -log(DATA1.2$pvalue, base = 10)*sign(DATA1.2$log2FC.x))
>DATA2_ordered <- cbind(data.frame(DATA2.2$geneID,stringsAsFactors =TRUE), -log(DATA2.2$pval, base = 10)*sign(DATA2.2$log2FC.y))

#remove any duplicates and rename the rows
>DATA1_order_unique<-DATA1_ordered[!duplicated(DATA1_ordered$DATA1.2.geneID), ]
>DATA2_order_unique<-DATA2_ordered[!duplicated(DATA2_ordered$DATA2.2.geneID), ]
>rownames(DATA1_order_unique) <- DATA1_order_unique$DATA1.2.geneID
>rownames(DATA2_order_unique) <- DATA2_order_unique$DATA2.2.geneID

#RRHO2
RRHO2(DATA1_order_unique, DATA2_order_unique, labels=c("mouse","human"), plots=TRUE, outputdir="~/OUTPUTDIR/")
```

WGNCA

```
library(WGCNA)
options(stringsAsFactors = FALSE)

datExpr0 = as.data.frame(t(S1819[, -c(1)]))
#datExpr0 <- as.data.frame(apply(datExpr0, 2, as.numeric))
names(datExpr0) = S1819$EnsembleGeneID
rownames(datExpr0) = names(S1819)[-c(1)]

gsg = goodSamplesGenes(datExpr0, verbose = 3)
gsg$allOK

# If the last statement returns TRUE, all genes have passed the cuts. If not, we remove the o ending genes and samples from the data

if (!gsg$allOK)
{
  # Optionally, print the gene and sample names that were removed:
  if (sum(!gsg$goodGenes)>0)
    printFlush(paste("Removing genes:", paste(names(datExpr0)[!gsg$goodGenes], collapse = ", ")));
```

```
  if (sum(!gsg$goodSamples)>0)
    printFlush(paste("Removing samples:", paste(rownames(datExpr0)[!gsg$goodSamples],
collapse = ", ")));
  # Remove the offending genes and samples from the data:
  datExpr0 = datExpr0[gsg$goodSamples, gsg$goodGenes]
}

# Next we cluster the samples (in contrast to clustering genes that will come later) to see if
there are any obvious outliers

sampleTree = hclust(dist(datExpr0), method = "average")
# Plot the sample tree: Open a graphic output window of size 12 by 9 inches
# The user should change the dimensions if the window is too large or too small.
sizeGrWindow(12,9)
#pdf(file = "Plots/sampleClustering.pdf", width = 12, height = 9);
par(cex = 0.6);
par(mar = c(0,4,2,0))
plot(sampleTree, main = "Sample clustering to detect outliers", sub="", xlab="", cex.lab = 1.5,
     cex.axis = 1.5, cex.main = 2)

#if outlier
# Plot a line to show the cut
abline(h = 65, col = "red");
# Determine cluster under the line
clust = cutreeStatic(sampleTree, cutHeight = 65, minSize = 10)
table(clust)
# clust 1 contains the samples we want to keep.
keepSamples = (clust==1)
datExpr = datExpr0[keepSamples, ]
nGenes = ncol(datExpr)
nSamples = nrow(datExpr)

#if no outlier
datExpr = datExpr0

# import behavior file
library(readxl)

# Form a data frame analogous to expression data that will hold the clinical traits.
Samples = rownames(datExpr);
traitRows = match(Samples, allTraits$subject);
datTraits = allTraits[traitRows, -1];
#datTraits <- as.data.frame(apply(allTraits, 2, as.numeric))
rownames(datTraits) = allTraits$subject

#We now have the expression data in the variable datExpr, and the corresponding clinical
traits in the variable datTraits. Before we continue with network construction and module
detection, we visualize how the clinical traits relate to the sample dendrogram.
# Re-cluster samples
sampleTree2 = hclust(dist(datExpr), method = "average")
```

```
# Convert traits to a color representation: white means low, red means high, grey means
missing entry
traitColors = numbers2colors((datTraits), signed = FALSE);
# Plot the sample dendrogram and the colors underneath.
plotDendroAndColors(sampleTree2, traitColors,
                    groupLabels = names(datTraits),
                    main = "Sample dendrogram and trait heatmap")

#save input
save(datExpr, datTraits, file = "men_homol_061020-01-dataInput.RData")
rm(allTraits,datExpr,datExpr0,datTraits,gsg,S1819,sampleTree,sampleTree2,traitColors,clust
,keepSamples,nGenes, nSamples,Samples,traitRows)

#Automatic block-wise network construction and module detection for large dataset
lnames = load(file = "mouse_homol_DATE-01-dataInput.RData")

#Choosing the soft-thresholding power: analysis of network topology
powers = c(c(1:10), seq(from = 12, to=20, by=2))
sft = pickSoftThreshold(datExpr, powerVector = powers, verbose = 5)
write.csv(sft$fitIndices, file="power_table.csv")

#Scale independance and mean connectivity
sizeGrWindow(9, 5)
par(mfrow = c(1,2))
cex1 = 0.9

# Scale-free topology fit index as a function of the soft-thresholding power
plot(sft$fitIndices[,1], -sign(sft$fitIndices[,3])*sft$fitIndices[,2],
     xlab="Soft Threshold (power)",ylab="Scale Free Topology Model Fit,signed
R^2",type="n",
     main = paste("Scale independence"))
text(sft$fitIndices[,1], -sign(sft$fitIndices[,3])*sft$fitIndices[,2],
     labels=powers,cex=cex1,col="red")

# this line corresponds to using an R^2 cut-off of h
abline(h=0.80,col="red")

# Mean connectivity as a function of the soft-thresholding power
plot(sft$fitIndices[,1], sft$fitIndices[,5],
     xlab="Soft Threshold (power)",ylab="Mean Connectivity", type="n",
     main = paste("Mean connectivity"))
text(sft$fitIndices[,1], sft$fitIndices[,5], labels=powers, cex=cex1,col="red")

#choose the power that is at the beginning of the flattening of the curve
bwnet = blockwiseModules(datExpr, maxBlockSize = 10000,
                         power = 8, TOMType = "unsigned", minModuleSize = 30,
                         reassignThreshold = 0, mergeCutHeight = 0.25,
                         numericLabels = TRUE,
                         saveTOMs = TRUE,
                         saveTOMFileBase = "men_homol-blockwise",
                         verbose = 3)
```

```
table(bwnet$colors)
sizeGrWindow(12, 9)
mergedColors = labels2colors(bwnet$colors)

plotDendroAndColors(bwnet$dendrograms[[1]], mergedColors[bwnet$blockGenes[[1]]],
                    "Module colors",
                    dendroLabels = FALSE, hang = 0.03,
                    addGuide = TRUE, guideHang = 0.05)

plotDendroAndColors(bwnet$dendrograms[[2]], mergedColors[bwnet$blockGenes[[2]]],
                    "Module colors",
                    dendroLabels = FALSE, hang = 0.03,
                    addGuide = TRUE, guideHang = 0.05)

# if more than 2 block repeat again

#save module assigment and module eigengene
moduleLabels = bwnet$colors
moduleColors = labels2colors(bwnet$colors)
MEs=bwnet$MEs
geneTree=bwnet$dendrograms
save(MEs, moduleLabels, moduleColors, geneTree,
     file = "men_homol_DATE-02-networkConstruction-bw.RData")

#Relating modules to external clinical traits
#Quantifying module-trait association
nGenes = ncol(datExpr)
nSamples = nrow(datExpr)
MEs0 = moduleEigengenes(datExpr, moduleColors)$eigengenes
MEs = orderMEs(MEs0)
moduleTraitCor = cor(MEs, datTraits, use = "p")
moduleTraitPvalue = corPvalueStudent(moduleTraitCor, nSamples)

#creating heatmap for module-trait association
sizeGrWindow(12,9)
# Will display correlations and their p-values
textMatrix = paste(signif(moduleTraitCor, 2), "\n(",signif(moduleTraitPvalue, 1), ")", sep = "");
dim(textMatrix) = dim(moduleTraitCor)
par(mar = c(6, 8.5, 3, 3));

# Display the correlation values within a heatmap plot
labeledHeatmap(Matrix = moduleTraitCor,
               xLabels = names(datTraits),
               yLabels = names(MEs),
               ySymbols = names(MEs),
               colorLabels = FALSE,
               colors = blueWhiteRed(50),
               textMatrix = textMatrix,
               setStdMargins = FALSE,
               cex.text = 0.45,
```

```
            zlim = c(-1,1),
            main = paste("Module-trait relationships"))

# Define variable weight containing the weight column of datTrait
group = as.data.frame(datTraits$groupe);
names(group) = "group"
# names (colors) of the modules
modNames = substring(names(MEs), 3)

geneModuleMembership = as.data.frame(cor(datExpr, MEs, use = "p"))
MMPvalue = as.data.frame(corPvalueStudent(as.matrix(geneModuleMembership),
nSamples))
names(geneModuleMembership) = paste("MM", modNames, sep="")
names(MMPvalue) = paste("p.MM", modNames, sep="")
geneTraitSignificance = as.data.frame(cor(datExpr, group, use = "p"))
GSPvalue = as.data.frame(corPvalueStudent(as.matrix(geneTraitSignificance), nSamples))
names(geneTraitSignificance) = paste("GS.", names(group), sep="")
names(GSPvalue) = paste("p.GS.", names(group), sep="")

#load file containing different Gene ID
annot <- geneAnnotation

dim(annot)
names(annot)
probes = names(datExpr)
probes2annot = match(probes, annot$EnsembleGeneID)
# The following is the number or probes without annotation:
sum(is.na(probes2annot))
# Should return 0

# Create the starting data frame
geneInfo0 = data.frame(EnsemblGeneID = probes,
                  geneSymbol = annot$geneID[probes2annot],
                  moduleColor = moduleColors,
                  geneTraitSignificance,
                  GSPvalue)
# Order modules by their significance for weight
modOrder = order(-abs(cor(MEs, group, use = "p")));
# Add module membership information in the chosen order
for (mod in 1:ncol(geneModuleMembership))
{
  oldNames = names(geneInfo0)
  geneInfo0 = data.frame(geneInfo0, geneModuleMembership[, modOrder[mod]],
                   MMPvalue[, modOrder[mod]]);
  names(geneInfo0) = c(oldNames, paste("MM.", modNames[modOrder[mod]], sep=""),
                  paste("p.MM.", modNames[modOrder[mod]], sep=""))
}
# Order the genes in the geneInfo variable first by module color, then by
geneTraitSignificance
geneOrder = order(geneInfo0$moduleColor, -abs(geneInfo0$GS.group));
geneInfo = geneInfo0[geneOrder, ]
```

```
rownames(geneInfo) <- NULL
write.csv2(geneInfo, file = "geneInfo.csv")
gene_module_men=geneInfo[,c(1,3)]
write.csv2(gene_module_men, file = "gene_module_men.csv")

# module overlap
gene_module_men <- read.csv("gene_module_human.csv")
gene_module_mouse <- read.csv("gene_module_mouse.csv")
h_dedup <- gene_module_men[!duplicated(gene_module_men$EnsemblGeneID),]
m_dedup <- gene_module_mouse[!duplicated(gene_module_mouse$EnsemblGeneID),]
labels1 <- h_dedup$moduleColor
labels2 <- m_dedup$moduleColor.x
fischer <- overlapTable(labels1, labels2, na.rm=TRUE, ignore=NULL)

#Interfacing network analysis with other data such as functional annotation and gene
ontology

probes = names(datExpr)
probes2annot = match(probes, annot$EnsembleGeneID)

allLLIDs = annot$entrezgene_id[probes2annot]

GOenr = GOenrichmentAnalysis(moduleColors, allLLIDs, organism = "human", nBestP = 10)

tab = GOenr$bestPTerms[[4]]$enrichment

write.csv2(tab, file = "GOEnrichmentTable.csv", quote = TRUE, row.names = FALSE)
```
