# Supplementary material for: The basolateral amygdala-anterior cingulate pathway contributes to depression-like behaviors and comorbidity with chronic pain behaviors in male mice
Source: Nat Commun. 2023 Apr 17;14:2198. doi: 10.1038/s41467-023-37878-y (PMC10110607; doi:10.1038/s41467-023-37878-y)
Supplement: Supplementary file 3 — Description of Additional Supplementary Files [file 41467_2023_37878_MOESM3_ESM.pdf]

Description of Additional Supplementary Files

File Name: Supplementary Data 1
Description: Differentially expressed genes in control vs. stimulated mice (Wald test with Benjamini and Hochberg correction for multiple testing), and associated gene ontology analysis (performed with WEBGESTALT, Hypergeometric test with Benjamini-Hochberg multiple testing correction).

File Name: Supplementary Data 2
Description: Gene ontology analysis of mice and men overlapping genes (see Extended Data Figure 5c), identified using RRHO2 (performed with WEBGESTALT, Hypergeometric test with Benjamini-Hochberg multiple testing correction).

File Name: Supplementary Data 3
Description: Gene ontology analysis of mice and human overlapping genes (women and men pooled; see Extended Data Figure 6a), identified using RRHO2 (performed with WEBGESTALT, Hypergeometric test with Benjamini-Hochberg multiple testing correction).

File Name: Supplementary Data 4
Description: Fisher’s exact test (two-sided) results for overlaps among mice and men modules identified using WGCNA.

File Name: Supplementary Data 5
Description: Fisher’s exact test (two-sided) results for overlaps among mice and human (women and men) modules identified using WGCNA.

File Name: Supplementary Data 6
Description: Description of mice cohorts used in Fig.4.
