# Supplementary material for: The basolateral amygdala-anterior cingulate pathway contributes to depression-like behaviors and comorbidity with chronic pain behaviors in male mice
Source: Nat Commun. 2023 Apr 17;14:2198. doi: 10.1038/s41467-023-37878-y (PMC10110607; doi:10.1038/s41467-023-37878-y)
Supplement: Supplementary file 10 — Reporting Summary [file 41467_2023_37878_MOESM10_ESM.pdf]

nature portfolio

Corresponding author(s): Ipek Yalcin

Last updated by author(s): 14/03/23

Reporting Summary

Nature Portfolio wishes to improve the reproducibility of the work that we publish. This form provides structure for consistency and transparency in reporting. For further information on Nature Portfolio policies, see our Editorial Policies and the Editorial Policy Checklist.

Statistics

For all statistical analyses, confirm that the following items are present in the figure legend, table legend, main text, or Methods section.

n/a	Confirmed	
☐	☒	The exact sample size (*n*) for each experimental group/condition, given as a discrete number and unit of measurement
☐	☒	A statement on whether measurements were taken from distinct samples or whether the same sample was measured repeatedly
☐	☒	The statistical test(s) used AND whether they are one- or two-sided<br>*Only common tests should be described solely by name; describe more complex techniques in the Methods section.*
☒	☐	A description of all covariates tested
☐	☒	A description of any assumptions or corrections, such as tests of normality and adjustment for multiple comparisons
☐	☒	A full description of the statistical parameters including central tendency (e.g. means) or other basic estimates (e.g. regression coefficient) AND variation (e.g. standard deviation) or associated estimates of uncertainty (e.g. confidence intervals)
☐	☒	For null hypothesis testing, the test statistic (e.g. *F*, *t*, *r*) with confidence intervals, effect sizes, degrees of freedom and *P* value noted<br>*Give P values as exact values whenever suitable.*
☒	☐	For Bayesian analysis, information on the choice of priors and Markov chain Monte Carlo settings
☒	☐	For hierarchical and complex designs, identification of the appropriate level for tests and full reporting of outcomes
☐	☒	Estimates of effect sizes (e.g. Cohen's *d*, Pearson's *r*), indicating how they were calculated

Our web collection on statistics for biologists contains articles on many of the points above.

Software and code

Policy information about availability of computer code

Data collection	MRI data acquisition was performed via Paravision 6.1 software (Bruker, Biospin, Germany) available on the MRI system.
Data analysis	MRI data processing was carried out using: MRtrix3, Advanced Normalization Tools (ANTS) and Statistical Parametric Mapping12 (SPM12)<br>All the tools used for RNA-sequencing analysis have already been published and are freely available. These notably include: STAR version 2.5.3a for RNA-Seq alignment, htseq-count version 0.6.1p1 for gene quantification; DESeq2 version 1.22.1 Bioconductor and R package for gene expression analysis; Caleb Huo script (https://github.com/RRHO2/RRHO2) for RRHO2 analysis, GSEA version 4.2.3 for Gene Set Enrichment Analysis and the WGCNA_1.70-3 R package for WGCNA analysis. Graphs were prepared by using GraphPad Prism v9.0 software. Statistical analyses were performed in GraphPas Prism v9.0 software.

For manuscripts utilizing custom algorithms or software that are central to the research but not yet described in published literature, software must be made available to editors and reviewers. We strongly encourage code deposition in a community repository (e.g. GitHub). See the Nature Portfolio guidelines for submitting code & software for further information.

Data

Policy information about availability of data

All manuscripts must include a data availability statement. This statement should provide the following information, where applicable:

- Accession codes, unique identifiers, or web links for publicly available datasets
- A description of any restrictions on data availability
- For clinical datasets or third party data, please ensure that the statement adheres to our policy

Source data files for all data presented in graphs within the figures are provided with this paper. Raw and processed human data reported in this study using post-mortem brain tissue from the ACC are publicly available on NCBI's GEO Datasets website , via the Gene Expression Omnibus accession number "GSE151827" (https://www.ncbi.nlm.nih.gov/geo/query/acc.cgi?acc=GSE151827; samples: GSM5026548-97). Raw and processed mouse RNA-Sequencing data related to transcriptional signature of neuropathic pain, published by Dai et al and used in the present study for comparison with optogenetic activation of the BLA-ACC pathway (Extended Data Figure 8) are publicly available via the Gene Expression Omnibus accession number "GSE197233" (https://www-ncbi-nlm-nih-gov.insb.bib.cnrs.fr/geo/query/acc.cgi?acc=GSE197233). Finally, raw and processed RNA-sequencing data regarding transcriptional effects, in the ACC, of optogenetic activation of the BLA-ACC pathway, are publicly available via the Gene Expression Omnibus accession number "GSE227159".

Human research participants

Policy information about studies involving human research participants and Sex and Gender in Research.

Reporting on sex and gender	Sex is reported in the manuscript and taken into consideration during the analysis.
Population characteristics	Brain tissue was obtained from the Douglas Bell Canada Brain Bank (DBCBQ; Douglas Mental Health Institute, Verdun, Québec; www.douglasbrainbank.ca). All subjects were Caucasians of French–Canadian descent, a population with a well identified founder effect. Inclusion criteria for both cases and controls were the following: the subject had to be Caucasian and of French Canadian origin and the subject had to die suddenly without prolonged agonal state. Tissue dissection was performed by histopathologists using reference neuroanatomical maps. Information concerning psychiatric history and socio-demographics was obtained by way of psychological autopsies performed by trained clinicians with the informants best acquainted with the deceased. Diagnoses were obtained using DSM-IV criteria by means of SCID-I interviews adapted for psychological autopsies. Control (C) and major depressive disorder (MDD) groups were matched for age, gender, post mortem interval and RNA integrity values, all meaningful covariates.
Recruitment	The Douglas-Bell Canada Brain Bank (www.douglasbrainbank.ca) collects brain tissue in collaboration with the Montréal coroner office as described in the text. Psychological autopsies were performed by trained clinicians on both controls and cases, with the informants best-acquainted with the deceased, as validated by our group and others. Diagnoses were assigned based on DSM IV criteria. Characterization of early-life histories was based on adapted Childhood Experience of Care and Abuse (CECA) interviews assessing experiences of sexual and physical abuse, psychological abuse, as well as neglect, and for which scores from siblings are highly concordant. We considered as severe early-life adversity reports of non-random major physical and/or sexual abuse during childhood (up to 15 years). Only cases with the maximum severity ratings of 1 and 2 were included. This information was then complemented with medical charts and coroner records. Ethical approval was obtained from the Institutional Review Board of the Douglas Mental Health University Institute. Written informed consent was obtained from the families of each of the deceased subjects prior to inclusion in the study.
Ethics oversight	This study was approved by IRB (Douglas Mental Health Institute Research Ethics Board), and signed informed consent was obtained from next of kin.

Note that full information on the approval of the study protocol must also be provided in the manuscript.

Field-specific reporting

Please select the one below that is the best fit for your research. If you are not sure, read the appropriate sections before making your selection.

☒ Life sciences ☐ Behavioural & social sciences ☐ Ecological, evolutionary & environmental sciences

For a reference copy of the document with all sections, see nature.com/documents/nr-reporting-summary-flat.pdf

Life sciences study design

All studies must disclose on these points even when the disclosure is negative.

Sample size	Our sample sizes are similar to those reported in previous publications (Barthas et al., 2017; Yalcin et al., 2011; Sellmeijer et al., 2018). Number of animals, number of slices, number of cells used in this study can be found in the figure legends of corresponding figures.
Data exclusions	No statistical outlier was removed.
Replication	All experiment were suscessully replicated at least once with several animals. The precise numbers are given in the figure legends.

Randomization	Mice were allocated to experimental groups randomly. For the electrophysiological recordings, cells were also selected randomly. For the immunohistochemistry, coronal sections (40micrometer) were serially collected and sections of every 160 micrometer are used.
Blinding	Detailed information concerning the blinding can be found in the methods section.

Reporting for specific materials, systems and methods

We require information from authors about some types of materials, experimental systems and methods used in many studies. Here, indicate whether each material, system or method listed is relevant to your study. If you are not sure if a list item applies to your research, read the appropriate section before selecting a response.

Materials & experimental systems

n/a	Involved in the study	
☐	☒	Antibodies
☐	☒	Eukaryotic cell lines
☒	☐	Palaeontology and archaeology
☐	☒	Animals and other organisms
☒	☐	Clinical data
☒	☐	Dual use research of concern

Methods

n/a	Involved in the study	
☒	☐	ChIP-seq
☒	☐	Flow cytometry
☐	☒	MRI-based neuroimaging

Antibodies

Antibodies used	PDGFRa; RandD systems, 1:200, AF1062<br>Olig2; Merck-Millipore, 1:200, AB9610<br>c-Fos: Synaptic System, 1:1000, 226-003 and Santa Cruz Biotechnology, E1008
Validation	PDGFRa see: https://www.rndsystems.com/products/mouse-pdgf-ralpha-antibody_af1062<br>Olig2 see: https://www.emdmillipore.com/US/en/product/Anti-Olig-2-Antibody,MM_NF-AB9610<br>c-Fos see: https://sysy.com/product-factsheet/SySy_226008<br>All the primary antibodies that are extensively used for IHC were used in non-living tissue for immunohistochemistry.

Eukaryotic cell lines

Policy information about cell lines and Sex and Gender in Research

Cell line source(s)	HEK 293T cells, ATCC CRL-3216 for the sh efficiency test, HEK 293T/17 ATCC CRL-11268 for rAAV production. Human Embrionic Kidney cells, laboratory adapted, no primary cells or patient derived cells.
Authentication	none
Mycoplasma contamination	This cell line was tested negative for mycoplasma contamination (test made by the cell culture service of the institute).
Commonly misidentified lines (See ICLAC register)	No commonly misidentified cell lines were used in this study.

Animals and other research organisms

Policy information about studies involving animals; ARRIVE guidelines recommended for reporting animal research, and Sex and Gender in Research

Laboratory animals	Mus musculus, C57BL/6J, 6 to 19 weeks. Mice are group-housed with a maximum of 5 animals per cage and kept under a reversed 12 h light/dark cycle. After the optic fiber implantation, animals were single housed to avoid possible damage to the implant. Room temperature 23-24°C and humidity around 50%.
Wild animals	The study did not involved wild animals
Reporting on sex	Only male mice were used in this study
Field-collected samples	This study does not involve samples collected from the field
Ethics oversight	The study protocols were approved by the local ethical committee of the University of Strasbourg (CREMEAS, APAFIS8183-2016121317103584)

Note that full information on the approval of the study protocol must also be provided in the manuscript.

Magnetic resonance imaging

Experimental design

Design type	Resting state fMRI, anatomical T2 weighted and diffusion tensor imaging
Design specifications	no tasks involved
Behavioral performance measures	*State number and/or type of variables recorded (e.g. correct button press, response time) and what statistics were used to establish that the subjects were performing the task as expected (e.g. mean, range, and/or standard deviation across subjects).*

Acquisition

Imaging type(s)	MRI, resting state fMRI, diffusion tensor imaging
Field strength	7 Tesla
Sequence & imaging parameters	RsfMRI: sequence = single shot GE-EPI, 147×59, TE/TR=15ms/2000ms, 500 image volumes, 0.14×0.23×0.5mm3 resolution, acquisition time = 16min.<br>T2 weigted: sequence = RARE, 0.08×0.08×0.4mm3 resolution, TE/TR=40ms/4591ms; 48 slices, 0.4mm slice thickness, interlaced sampling, RARE factor of 8, 4 averages; an acquisition matrix of 256×256 and FOV of 2.12×2cm2.<br>Diffusion tesnor imaging: sequence = 4-shot DTI-EPI, TE/TR=24ms/3000ms, 8 averages, 45 directions, b-factor = 0s/mm^2 and 1000s/mm^2, 30 axial slices with 0.5mm thickness, FOV = 1.9×1.6cm2, acquisition matrix = 190×160, resolution = 667 0.1×0.1×0.5mm3
Area of acquisition	whole mouse brain

Diffusion MRI ☒ Used ☐ Not used

Parameters: 45 directions, b factor = 0s/mm^2 and 1000s/mm^2; single shell, no cardiac gating

Preprocessing

Preprocessing software	MRI data processing was carried out using: MRtrix3, Advanced Normalization Tools (ANTS) and Statistical Parametric Mapping12 (SPM12)
Normalization	MRI data was spatially normalised on the Allen Mouse Brain Atlas
Normalization template	Allen Mouse Brain Atlas template
Noise and artifact removal	Ghosts artifacts removal was performed
Volume censoring	A whole brain mask was used for the resting state fMRI data analysis

Statistical modeling & inference

Model type and settings	General linear model
Effect(s) tested	Effect(s) tested : variation of diffusion scalar metrics between wild-type and knock-out mice / correlation between diffusion scalar metrics and behavioral tests

Specify type of analysis: ☐ Whole brain ☐ ROI-based ☒ Both

Anatomical location(s): Seed analysis was performed on the rsfMRI data, using as seed the anterior cingulate cortex

Statistic type for inference (See Eklund et al. 2016)	two-samples t-test / correlation
Correction	rsfMRI: $p<0.05$; FDR cluster corrected; diffusion MRI - $p<0.05$ FDR cluster corrected; for diffusion to behavior test correlation: $p<0.001$, uncorr

Models & analysis

n/a	Involved in the study
☐	☒ Functional and/or effective connectivity
☐	☐ Graph analysis
☐	☐ Multivariate modeling or predictive analysis

Functional and/or effective connectivity	for resting state fMRI: Pearson correlation
Graph analysis	*Report the dependent variable and connectivity measure, specifying weighted graph or binarized graph, subject- or group-level, and the global and/or node summaries used (e.g. clustering coefficient, efficiency, etc.).*
Multivariate modeling and predictive analysis	*Specify independent variables, features extraction and dimension reduction, model, training and evaluation metrics.*
